# Immersive virtual reality health games: a narrative review of game design

**DOI:** 10.1186/s12984-020-00801-3

**Published:** 2021-02-11

**Authors:** Gordon Tao, Bernie Garrett, Tarnia Taverner, Elliott Cordingley, Crystal Sun

**Affiliations:** 1 Graduate Programs in Rehabilitation Science, Faculty of Medicine, University of British Columbia, Vancouver, BC Canada; 2 School of Nursing, University of British Columbia, Vancouver, BC Canada; 3 Faculty of Science, University of British Columbia, Vancouver, BC Canada

**Keywords:** Virtual reality, Head mounted display, HMD, Health game, Exergame, Rehabilitation games

## Abstract

**Background:**

High quality head-mounted display based virtual reality (HMD-VR) has become widely available, spurring greater development of HMD-VR health games. As a behavior change approach, these applications use HMD-VR and game-based formats to support long-term engagement with therapeutic interventions. While the bulk of research to date has primarily focused on the therapeutic efficacy of particular HMD-VR health games, how developers and researchers incorporate best-practices in game design to achieve engaging experiences remains underexplored. This paper presents the findings of a narrative review exploring the trends and future directions of game design for HMD-VR health games.

**Methods:**

We searched the literature on the intersection between HMD-VR, games, and health in databases including MEDLINE, Embase, CINAHL, PsycINFO, and Compendex. We identified articles describing HMD-VR games designed specifically as health applications from 2015 onwards in English. HMD-VR health games were charted and tabulated according to technology, health context, outcomes, and user engagement in game design.

**Findings:**

We identified 29 HMD-VR health games from 2015 to 2020, with the majority addressing health contexts related to physical exercise, motor rehabilitation, and pain. These games typically involved obstacle-based challenges and extrinsic reward systems to engage clients in interventions related to physical functioning and pain. Less common were games emphasizing narrative experiences and non-physical exercise interventions. However, discourse regarding game design was diverse and often lacked sufficient detail. Game experience was evaluated using primarily ad-hoc questionnaires. User engagement in the development of HMD-VR health games primarily manifested as user studies.

**Conclusion:**

HMD-VR health games are promising tools for engaging clients in highly immersive experiences designed to address diverse health contexts. However, more in-depth and structured attention to how HMD-VR health games are designed as game experiences is needed. Future development of HMD-VR health games may also benefit from greater involvement of end-users in participatory approaches.

## Background

In recent years, there has been a rapid growth in the reported use of virtual reality (VR) in the treatment of a variety of clinical conditions, such acute and chronic pain management [1–3], phobias, anxiety and other disorders or health conditions [4–13]. VR as a concept has come to include a wide range of digital technology applications where the user perceives and interacts with a computer-generated virtual environment, whether through a traditional 2-dimensional (2D) display, a projected display paired with 3D glasses, or a head-mounted display (HMD). Given such diverse types of display technologies, comparisons may not be reliable, and so this review is focused on HMD-VR technology applications. Similarly, general software content of HMD-VR ranges from artistic virtual experiences, to simulations, to games. This review focuses on games designed specifically as health applications, or HMD-VR health games.

### Advances in HMD-VR technology

Research in the 1980′s with early computer graphics explored the potential for computer-generated VR experiences with HMDs. At the time, computer processing power was very limited while HMDs were unreliable and cost tens of thousands of dollars. More recently, commercially available computer and graphics technology has become capable of rendering realistic high resolution 3D imagery in real-time. The current level of technology is far removed from devices we were using in research even five years ago (such as the Oculus Rift Development Kit 2). In 2005, basic cloth simulation was hailed as showpiece technology, whilst modern 3D rendering cards with integrated physics support can simulate individual human hair movement in complex lighting situations. This technological progress has made modern VR devices much more immersive, providing a much better sense of presence for demanding VR gaming users, and more importantly, less prone to technical problems than their early counterparts. Whilst the typical cost of a high-end consumer HMD-VR set-up remains high for individual consumers ($2000 or more, including gaming computer and headset system), this generation of technology has seen substantial use in research settings [14]. Moreover, VR technology companies have released more accessible, affordable, and easy to use HMDs, e.g. Oculus Quest (Facebook, Menlo Park, USA). These advancements will support greater accessibility of HMD-VR, including HMD-VR health applications.

### Applying gaming and HMD-VR in health

The key element in these HMD-VR health applications is the use of immersive media content to engage the users in a stimulating interactive experience, where they feel a sense of presence in a different and novel environment [14, 15]. This is quite different from two-dimensional computer based gaming implementations. Presence here, refers to the sense of actually being within an environment that is generated by technological means [14, 16–18]. It involves the participant as a co-constructor of the experience, and this concept is well-established in computer science [14]. Although third-person perspectives have been used and discussed in VR gaming experiences, this has not been a major focus for health game application developers, as the potential for VR to engender a sense of first-person presence represents one of its main attractions [19, 20]. Hence, this review has focused on HMD VR implementations, as the most common and rapidly developing field in VR health gaming applications.

The typical content takes the form of either a game or a purposeful, yet non-gaming, first-person virtual experience. A common application of the latter is seen in exposure therapy whereby patients with phobias are presented with the source of their anxiety or fear in a controlled and graded manner in order to help them overcome their distress response [21]. Another example is Virtual Meditative Walk where users follow a guided meditation to reduce anxiety and chronic pain. In this virtual environment, EEG biofeedback of entering relaxed mindful state causes mist to appear in the virtual forest [22]. Alternatively, off-the-shelf commercial HMD-VR games may be used as novel adjunctive interventions. Exercise-based games such as Audioshield [23] and Beat Saber [24] are rhythm-based HMD-VR games where players have to physically move their bodies to play. Beyond cardiovascular exercise, such games are of therapeutic interest for their involvement of psychomotor skills and even cognitive skills. Yet, the principal appeal behind using games for therapy, whether HMD-based or not, has been their potential to support sustained adherence to therapy [27–29]. As such, game-based therapy may be viewed as an approach to behavior change [30]. However, off-the-shelf commercial HMD-VR games are limited in their application. As they are designed for general able-bodied audiences, they may not be suitable for health contexts involving physical or cognitive limitations.

Another approach has involved developing games specifically as health applications [31–33]. For example, rehabilitation games for stroke recovery have seen technology implementations pairing motion capture technology with both 2D televisions and HMDs [34]. Health games are usually designed to address limitations in body functions or mental health, build specific skills, or promote positive behaviors in the player. They often involve combining elements such as puzzles, graded difficulty, matching patterns, repetitive exercises, exploring an environment, or simply providing a pleasing and distracting experience. An early example includes Hunter Hoffman’s SnowWorld, which used an older generation of HMD-VR technology. In SnowWorld, patients who had suffered burns throw snowballs at snowmen and penguins to reduce their pain [38, 39]. Such HMD-VR health games aim to combine the advantages of a fun and motivating game with clinically grounded approahces.

### Game design for HMD-VR health applications

Developing games as health applications using HMD-VR requires a broad intersection of theoretical and technical lenses. These include biomedical and psychosocial perspectives on health, computer and engineering technologies, human computer interaction theory, and ultimately game design. Several approaches to game design have been articulated by Salen and Zimmerman [35], Schell [36], and Fagerholt and Lorentzon [37]. Games may be fundamentally described as systems with defined rules, explicit objectives, and quantifiable outcomes, whereby interaction with these systems give rise to a playful experience [36]. These systems may be contrasted with other artefacts such as playgrounds, where there are no game rules, or training simulators, where play is not an important aspect of the experience. The ultimate design goal, according to Salen and Zimmerman, is an experience of meaningful play, which “occurs when the relationships between actions and outcomes in a game are both discernable and integrated into the larger context of the game” [35].

Considering Hunicke and colleague’s Mechanics-Dynamics-Aesthetics (MDA) framework [38], meaningful play can be understood through the Aesthetic experiences produced by a game such as fantasy, challenge, discovery, self-expression, etc. These Aesthetic experiences are the cumulative emotional and intellectual product of the game Dynamics: the various ways the player interacts, makes choices, and plays the game. Examples of Dynamics include resource management, time pressure, cooperation, collection, building, or other strategies. Underpinning the game Dynamics are the Mechanics that make up the objects and rules of the game. For example, Mechanics in Chess include the board layout, the different pieces, how the pieces can move and capture other pieces, and the objective and outcome of checkmating your opponent. With this conception of games in mind, we may consider the strengths and benefits of using HMD-VR for gameplay experiences and their impact in context of health applications. Primarily, HMD-VR greatly contributes to aesthetic experiences that rely on sensory immersion, e.g. discovery and fantasy.

Moreover, the quality of game design ultimately impacts stakeholder adoption of these games as health technology [39–41]. To this end, stakeholder engagement throughout the research and development process has also seen increasing emphasis [39, 42, 43]. Overall, HMD-VR health games represents a growing field, and some researchers are now beginning to compare the value of different hardware and software applications in this area [44]. Reviews including HMD-VR health games typically focus on efficacy in a specific context [2, 21, 45]. However, it is unclear how differences in the game design of HMD-VR health games impact their effectiveness compared to others. Often, broad comparisons are made in terms of very different systems, users, and assessment tools. We are gaining some ideas about the important elements in designing user accessible and effective HMD-VR experiences overall [14, 46, 47], but within this sphere, the value of good game design in the effectiveness of a health game remains relatively unexplored. Yet, it represents a significant part of the puzzle. Game design underpins the mechanisms by which HMD-VR health games influence patients as a behavior change approach. Therefore, an exploration of the application of game-design in HMD-VR is a significant and worthwhile area to explore further. To this end, we conducted a narrative review characterizing the current state and considerations of game design in HMD-VR health games.

## Methods

To explore game design in HMD-VR health games, a literature search focusing on the intersection between HMD-VR, games, and health was undertaken. Bibliographic health databases searched included MEDLINE, Embase, CINAHL, and PsycINFO. The engineering database Compendex with a search restriction to the “health” topic was also used. To narrow VR-related literature to only HMD-VR, the keywords: HMD, head mounted display, and virtual headset were used as search terms. For games, the keywords: game, gaming, and exergame were used. Search results were restricted to the English language. To capture the current generation of HMD-VR we also restricted results to those published from 2015 onwards. All search results arising from the Boolean “AND” of the HMD-VR and games search results were screened. Title and abstract screening included any articles mentioning VR or games in a health context. The full text screening criteria are provided in Table 1.

**Table 1:** Inclusion and exclusion criteria

Inclusion criteria	Exclusion criteria
Original articles describing the design of a digital application specifically for health contexts (e.g. reducing symptoms, recovering body or cognitive functions, or maintaining health) Use a HMD-VR as the display and interaction technology Describes the VR application as a game Available in the English language Published in peer reviewed academic or professional journals	Only used commercially available HMD-VR games designed for general audiences Opinion or narrative discussions that did not report on the use of a specific VR-based game Grey literature

HMD-VR health games were charted and tabulated according to the type of input technology implementation, health context (i.e. clinical population, condition, or type of intervention), outcomes used in the evaluation of the game, and user engagement employed during game design, if any. Given the wide range of approaches to game design and reporting, a narrative review appeared the most appropriate approach. Accordingly, we summarized each reviewed game according to the MDA framework and narratively described observed trends. Furthermore, we tabulated outcomes related to evaluation of identified games according to therapeutic outcomes, game experience, technology acceptance, cybersickness, and open feedback. Tabulated results were also integrated into each topic of discussion.

## Findings

The complete search was conducted on March 16, 2020 and identified 140 potentially relevant articles. Deduplication yielded 94 unique articles. Title and abstract screening identified 47 articles related to HMD-VR, games, and health. Upon full-text screening, 30 articles were identified as describing HMD-VR games designed specifically for health contexts [48–77]. One article referenced previous work with greater detail related to game design and was retrieved [57]. The 29 HMD-VR games are summarized in Table 2 and evaluation outcomes are characterized in Table 3. All data are provided together in Additional file 1.

**Table 2:** Identified HMD-VR health games and summary including technology implementation, health context, and game design. **M**echanics-**D**ynamics-**A**esthetics framework sumarizes game design. **Motivation** describes authors' explicit and specific association of theory and design with motivation or engagement beyond the ubiquitous “immersive games are motivating”. **Guidance **describes features that help instruct the player through the gameplay experience. **NS **refers to game design aspects **N**ot **S**pecified in the article, including common features such as rewards (points, badges, etc.) and narrative (story, theme, characters, etc.); this does not imply such elements are necessary game design features

Author	Year	Display and interaction technology	Intended health context and end-users	Game design summary
Gobron et al. [50]	2015	HMD + Lower Limb Haptic Robot	Lower limb rehabilitation for post-stroke patients; varied ages from kids to seniors	Four mini games with limited detail *Gate Crossing* M: Use feet to pilot spaceship through gates A: “Young space pilot learning to use a spaceship” *BikeRehab* M: Pedaling; pizza to deliver; obstacles in path; 2D side-scrolling world; score A: Be a great pizza delivery boy *BeTheBall* M: Leg press to move ball; “ghost” of best performance A: Racing through different outdoor environments as a ball, challenge to get highest score *PicsWalk* M: Walking motion to navigate; take photos A: Being a photographer walking through a beautiful forest full of wildlife and plants Motivation: Users motivated by playing games of interest NS: Narrative, Guidance
Shaw et al. [55]	2015	HMD + Skeletal Tracking + Cycle	Physical exercise for sedentary/overweight adults (implicit)	*Cycling Obstacle Course* M: Cycling forward and steering using body leaning through a branched course with more or fewer obstacles and reward objects; scoring based on speed, distance, and rewards collected; fixed number of lives D: Tensions between choosing speed vs avoiding obstacles and risk vs reward A: Overcoming a challenging obstacle course as fast as possible Motivation: Based on challenge, scoring, moment-moment choice in branches, non-repetitive play Guidance: Game explained by researchers NS: Narrative
Gromala et al. [48]	2016	HMD	Pain relief for persons with acute and chronic pain	*Mobius Floe* M: movement along a “rail” path, encounters with hostile “Neuron Trees” and helpful characters to interact with; varied projectiles; health packs and health points; shoot medication with different effects D: Defend against neuron trees in a “time-sensitive manner”; measured use of medication on Neuron Trees A: Be immersed and journey through an exciting snowy world; overcome challenges symbolizing pain experiences Motivation: The multisensory experience with high interactivity and thematic narrative helps to achieve immersive engagement; health points motivates focus on game tasks Guidance: In-game tutorial
Ijaz et al. [63]	2016	HMD + Skeletal Tracking + Cycle	Older vs younger adults and physical exercise	Two variants of a game: competitive, affiliative M: City environment with a map, compass, and using pedalling to navigate; competitive version involves guessing landmarks, scores based on performance, leaderboard, and levels; affiliative version involves audio descriptions of landmarks, taking photos, and a photo album D: Competitive: plan efficient route, predict landmarks, beat own/others’ scores, explore the city Affiliative: go to all landmarks, craft collection of photos A: Competitive: find your way in a strange city and be the best at it; Affiliative: Being a cycling tourist Motivation: Based on self-determination theory; competitive vs affiliative play Guidance: In-game tutorial NS: Narrative
Lv et al. [65]	2016	HMD + Microphone	Persons with dysphonia/presbyphonia	*Space Flight Game* M: Voice pitch and loudness controls elevation of a spaceship moving amongst planets as obstacles; score at end D: Avoid planets as long as possible A: Steering a 2D side-scrolling spaceship Guidance: Clinician guidance NS: Motivation, Narrative; vague mention of “game stories”
Thomas et al. [71]	2016	HMD + Skeletal Tracking (marker-based)	Movement promotion for persons with kinesiophobia	*Dodgeball Game* M: Player holding ball on dodgeball court against opposing computer players who launch balls at player at variable heights; incoming balls are coloured to indicate action to block or dodge; scoreboard D: Focused on reacting quickly to block or dodge, pre-positioning, beating own score A: Be an untouchable dodgeball player; improve at dodging and blocking Guidance: practice level to introduce mechanics NS: Motivation, Narrative
Tuveri et al. [49]	2016	HMD + Skeletal Tracking + Cycle	Physical exercise for a general audience (implicit)	*Rift-a-bike* M: Player pedals at varying speeds through three levels of a city bike tour: warm-up, exercise, cool-down; players rewarded with experience, skill, and karma points for performance and actions; challenge missions present objectives; Rabbit to follow; coins along cycling path D: Chase the rabbit and collect as many coins as possible. Complete missions A: A city cyclist taking on missions that make up the “game plot”; challenge of improving your score. Build your prize collection Motivation: Reward mechanics provide motivation for playing; rabbit is a persistent objective and relative position provides performance feedback NS: Guidance
Howes et al. [75]	2017	HMD + Skeletal Tracking	Strength and balance training for older adults at risk of falling	Several Minigames; limited detail NS: Motivation, Rewards, Narrative, Guidance
Nielsen et al. [67]	2017	HMD + Hand Motion Controllers	Persons with lower limb amputation and phantom limb pain	Two games; limited detail *Shape Game* M: foot positioning tracked by controller strapped to feet, incoming shapes, and points awarded for matching feet position to shape *Slingshot Game* M: Slingshot manipulated using feet, targets to shoot, points awarded NS: Motivation, Narrative, Guidance
Ambron et al. [58]	2018	HMD + Lower Limb IMU Sensors	Persons with lower limb amputation and phantom limb pain	Three games adapted: Chess, Checkers, and Quest for Fire *Quest for Fire* M: Boxes and other objects for overcoming obstacles in each level of a labyrinth D: Players could create the best routes for navigating through the labyrinth A: Escape the labyrinth Motivation: Authors used “rich virtual environments” to increase motivation NS: Rewards, Narrative, Guidance not specified
Avola et al. [56]	2018	HMD + Skeletal Tracking + Hand Tracking	Upper limb rehabilitation for persons with neuromotor impairments	*VRheab*, limited detail Some mechanics involving finger pinching and raising knees NS: Rewards, Narrative, Guidance
Caggianese et al. [53]	2018	HMD	Cognitive training for persons with Alzheimer’s disease and mild cognitive impairment	Activities of Daily Living based scenarios involving motion and memory tasks; limited detail Motivation: Customized difficulty level by clinician and encouragement to do tasks on their own Guidance: Clinician guided NS: Rewards, Narrative
Czub and Piskorz [59]	2018	HMD	Treatment of acute pain	M: Sphere avatar, steering, obstacle red sphere, reward white sphere D: Collect as many points as possible by hitting white spheres and avoiding red spheres A: Challenge of getting a high score Guidance: practice phase of experiment NS: Motivation, Narrative
Eisapour et al. [78]	2018	HMD + Hand Motion Controllers	Physical Exercise for persons with dementia	Untitled Exergame M: Interactions based on neck, shoulder, and arm movement; tasks involving objects to reach for and place; placing tasks adjustable for range of motion; farm-related objects D: Four scenarios: follow the butterfly with head; lift apple boxes; sort fruit; row the boat A: Doing calm things on a pleasant farm Guidance: Pre-recorded instructions for each scenario and task NS: Motivation, Rewards, Narrative
Huang et al. [61]	2018	HMD + Robotic Arm	Upper Limb Rehabilitation (Stroke)	*SpaceWar 3D* M: Control spaceship position; incoming meteorites; shoot missiles; enemy spaceships; score based on time and number of enemies shot down D: Dodge meteorites, target and shoot spaceships; tensions between avoiding collisions and targeting enemies A: Being an ace space combat pilot Motivation: Dynamic objects and audio as feedback that increases engagement NS: Guidance, Narrative
Lee and Kim [64]	2018	HMD	Binocularity stimulation for children with residual amblyopia	*Ice Cream Truck* M: Running kids; throwable ice cream; difficulty level D: Hit as many kids with ice cream as you can A: Feeding all the kids ice cream NS: Motivation, Rewards, Narrative, Guidance
Mihajlovic et al. [66]	2018	HMD	Neck Pain Rehabilitation	Untitled Exergame; multiple scenarios M: Track objects with head motion; butterfly target; net to catch butterfly; score based on caught butterflies and movement quality; different levels D: Catch as many butterflies as possible, quickly and accurately A: Be a butterfly catcher in realistic indoor environments Motivation: Realistic immersive environment with butterfly as objective and feedback NS: Narrative, Guidance
Piskorz and Czub [68]	2018	HMD	Acute Pediatric Pain and Stress	Untitled game based on “Multiple Object Tracking paradigm”; limited detail M: Flashing elements, scenes where flashed elements are located, head movement to control game; difficulty level D: Memorize and find the flashed elements Guidance: Two 10–15 min training scenes NS: Rewards, Narrative
Proffitt et al. [69]	2018	HMD + Skeletal Tracking	Stroke Motor Rehabilitation (Full body)	*Mystic Isle* M: Avatar; different coloured bottles of varied heights; bin and button for filling; garbage bags; conveyor belt D: Sort bottles into correct bins; fill bottles completely without overflowing; load bags onto hooks A: Become an efficient recycler NS: Motivation, Narrative, Guidance
Sisto et al. [54]	2018	HMD + Skeletal Tracking	Posture and Preventing Musculoskeletal Disorders	Untitled Serious Game; game and level design explicitly addressed M: Puzzle board with pegs and replaced gears; gears to be selected and placed using hand movements; movement quality scoring; game score based on completion time D: Solve puzzle by selecting correct gears; tension between minimize time vs movement quality A: Spaceship and robot theme; solving puzzles Motivation: balancing game difficulty and cognitive load; not to hard/easy for engagement NS: Narrative, Guidance
Dias et al. [60]	2019	HMD + Markerless Hand Tracking	Upper limb rehabilitation for post-stroke patients	*A Priest in the Air* M: Steer hot-air balloon using arm and wrist position/rotation; obstacles; stars to collect; branching paths; difficulty selection; different biome environments; King’s Castle at the end D: Travel through different environments to reach the King’s Castle; maximize stars/points collection A: Player vs environment challenge; challenge improving overall high score; soaring through the air in a hot air balloon travelling the world; story set by opening and closing cutscenes with characters Priest, Dog’s Priest, King Motivation: defined by short, mid, and long-term goals; adjustable game difficulty Guidance: Explicit usability consideration; tutorial videos; help icons in-game
Ijaz et al. [62]	2019	HMD + Cycle	Physical exercise for a general audience (implicit)	*Pokemon Ride* M: Navigation via pedalling/steering through city with map and different locations and levels; pokemon at different locations to catch; score based on pokemon type; physiological feedback D: Find all the pokemon A: Collection and accumulation; collecting together with others but individually *Balloon Shooter* M: Similar map/navigation; different weapons for shooting balloons placed around map; points for shooting balloons D: Keep shooting balloons to unlock levels A: Be a crackshot balloon hunter in the city Motivation: based on Self-Determination Theory Guidance: in-game tutorial NS: Narrative
Nehrujee et al. [76]	2019	HMD	Rehabilitation for patients with vestibular dysfunction	*Cardboard Break* M: Head movement pitch/yaw based on vestibular training; reticle to aim at cardboard boxes that randomly appear with varied distance from centre; score by moving reticle onto box; countdown timer; adaptive difficulty D: Gaze shift to box as quickly as possible A: Challenge of getting a high score *Follow Me* M: Continuously moving ball; trials; score based on head tracking ball; adaptive difficulty D: Track the ball with your head for as long as possible A: Challenge of getting a high score Motivation: Adaptive algorithm to adjust difficulty to achieve state of flow NS: Narrative, Guidance
Rossi et al. [70]	2019	HMD	Treatment for persons with Sensory Processing Disorders	*Imaginator*; two scenarios, four tracks, three activities M: Rail and cart moving; different coloured moving balls, letters, and numbers; clinician sets task objective; player uses gaze to make selections; score based on correct selections; variable difficulty D: Hit the correct moving ball; hit moving letters in correct pattern; select correct sum A: Pleasant roll on tracks in an outdoor park Guidance: Clinician guided NS: Narrative; recommended to have future character interaction
Tong et al. [51]	2019	HMD + Hand Motion Controllers	Physical exercise for persons with arthritis	*Lumapath* M: Motion-based gestures aimed at arthritis physical activity; ship to drive; different planets; various plants and life forms of rarity; floating patter of dots to follow with gestures; teleportation to move through world D: Explore the planets; discover new things in environment; draw shapes following dot patterns A: Take your personal ship to explore different planets Guidance: 10 min tutorial; thematic analysis revealed participants would like more guidance through the experience NS: Motivation (thematic analysis revealed exploration played major role in motivation), Rewards, Narrative
Yao and Kim [72]	2019	HMD + Cycle	Physical exercise for a general audience (implicit)	*VIR Zoom* (commercialized); limited detail NS: motivation, rewards, narrative, guidance
Yaramothu et al. [73]	2019	HMD	Vision therapy for convergence insufficiency	*VERVE (Virtual Eye Rotation Vision Exercises)*; limited detail M: convergence related mechanics NS: motivation, rewards, narrative, guidance
Xu et al. [20]	2020	HMD + Skeletal Tracking	Physical exercise for sedentary individuals	*GestureStar* M: blocks flying towards player; varied patterns of blocks requiring gestures of different workloads D: perform correct gesture just as block reaches you A: similar to commercially available rhythm and music games NS: motivation, rewards, narrative, guidance
*Barathi et al. [57]; Farrow et al. [52]	2018, 2019	HMD + Cycle	Physical exercise for sedentary individuals	Untitled Exergame explicitly designed according to MDA Framework M: cycling and steering; prompts and pattern of speed and resistance changes following HIIT (High Intensity Interval Training) with environmental changes; truck obstacles; best performance ghost; countdown timer D: maximize accomplishment in limited time; follow the HIIT protocol; race against yourself A: self improvement; cycling in traffic; nice sunny ride vs exciting night race Motivation: discussed in terms of intrinsic motivation and interactive feedforward method Guidance: instruction sheet about experiment and exergame NS: rewards, narrative

*These two articles describe the same HMD-VR health game. The outcomes were combined

**Table 3:** Outcomes from evaluation of identified games

Author	Year	None (system overview)	Therapeutic outcome	Game experience	Acceptance (usability, usefulness, etc.)	Cybersickness	Open feedback/ Interview
Gobron et al. [50]	2015	–	–	–	✓	–	–
Shaw et al. [55]	2015	–	✓	✓	✓	✓	✓
Gromala et al. [48]	2016	✓	–	–	–	–	–
Ijaz et al. [63]	2016	–	–	✓	✓	–	–
Lv et al. [65]	2016	✓	–	–	–	–	–
Thomas et al. [71]	2016	–	✓	–	–	–	–
Tuveri et al. [49]	2016	–	–	✓	✓	–	–
Howes et al. [75]	2017	–	–	–	✓ ✓	–	✓
Nielsen et al. [67]	2017	–	–	–	✓	–	–
Ambron et al. [58]	2018	–	✓	–	✓	–	–
Avola et al. [56]	2018	✓	–	–	–	–	–
Caggianese et al. [53]	2018	✓	–	–	–	–	–
Czub and Piskorz [59]	2018	–	✓	✓	✓	–	–
Eisapour et al. [78]	2018	–	✓	✓	✓	–	–
Huang et al. [61]	2018	–	✓	–	–	–	–
Lee and Kim [64]	2018	–	✓	–	–	–	–
Mihajlovic et al. [66]	2018	–	✓	✓	–	–	–
Piskorz and Czub [68]	2018	–	✓	–	–	–	–
Proffitt et al. [69]	2018	–	–	–	✓	–	✓
Sisto et al. [54]	2018	✓	–	–	–	–	–
Dias et al. [60]	2019	–	✓	–	✓	–	–
Ijaz et al [62]	2019	–	✓	✓	✓	–	–
Nehrujee et al. [76]	2019	–	✓	–	✓	✓ ✓	–
Rossi et al. [70]	2019	–	–	–	–	–	✓
Tong et al. [51]	2019	–	✓	✓	✓	–	✓
Yao and Kim [72]	2019	–	✓	✓	–	–	–
Yaramothu et al. [73]	2019	–	✓	–	–	–	–
Xu et al. [20]	2020	–	✓	✓	✓	✓	–
*Barathi et al. [57]; Farrow et al. [52]	2018; 2019	–	✓	✓	✓	–	✓

*These two articles describe the same HMD-VR health game. The outcomes were combined

### Health contexts and end-users

Of the HMD-VR health games reviewed, the most common health contexts included physical exercise, motor rehabilitation, and pain related conditions. Physical exercise games were aimed at both healthy adults and those with various health conditions. For example, Tuveri and colleagues designed Rift-a-bike to engage a general audience in cycling-based exercise [49] while Eisapour and colleagues focused on exercise for people with dementia [74]*.* Other health contexts ranged from addressing sensory disorders [70] to cognitive functioning [53]. Overall, these contexts are consistent with applications found in the broader health VR field, which includes non-game and non-HMD applications [78–80]. However, some key areas were not represented in the games reviewed. While HMD-VR applications have been used for mental health disorders such as phobias, depression, or body dysmorphia, those conditions have not seen HMD-VR health games designed specifically for them [14, 21]. Perhaps, for conditions such as mild depression, off-the-shelf commercial exergames (HMD-VR or otherwise) may suffice for improving certain aspects of function [81]. In such cases, a specifically developed HMD-VR health game may be unnecessary. Nevertheless, commercial game-based interventions may not always be an appropriate method of treatment delivery for these underrepresented health conditions. More research is necessary to understand how game playing and using HMD-VR, can support or conflict with therapies for such health conditions.

Conversely, HMD-VR games based on therapies involving physical movement were the dominant trend observed. In this context, the choice of interaction technology is particularly important. While HMD-VR provides the principle means of immersing the user in an environment, the quality of motion tracking determines the perceived realism of a user’s actions impacting the virtual environment [82]. This technology choice must also be aligned with the therapeutic modality, as each technology has its limitations. For example, skeletal tracking [83] is suitable for health applications involving gross movement over larger ranges of motion, as Sisto and colleagues used skeletal tracking for determining risk of musculoskeletal disorder during gameplay [54]. Conversely, hand tracking [84] is more suitable for exercises involving fine finger control, as seen in *VRheab* [56]. Therefore, the intersection of the health context with the interaction technology underpins the scope and technical limitations within which HMD-VR health games must be designed.

### Intersection of health context with interaction technology

HMD-VR health games have relied on the successful commercialization of economical VR headsets with suitable features for entertainment such as high-resolution displays, accessible development tools, and comfort. Moreover, the improved ability to track headsets with six degrees of freedom (i.e. up/down, left/right position and rotation about three perpendicular axes, pitch, roll and yaw) allows for more natural and immersive interaction with virtual environments. Compared to traditional 2-dimensional (2D) displays, playing games within the greater immersion of HMD-VR can lead to overall greater satisfaction, with emphasis on engrossment and creative freedom [85]. From the studies we reviewed, Xu and colleagues showed greater immersion, effort, flow, and affect in an HMD condition compared to a large 2D display during an exergame [77]. Other studies have demonstrated that HMD-VR heightens emotional responses such as happiness, anxiety, or surprise compared to 2D displays [47, 86]. Yet, the application of HMD-VR may also depend on the population and type of intervention. For example, Howes and colleagues [75] found their older adult participants preferred a 2D display over HMD during a balance training game. In an upper extremity focused game, post-stroke participants had mixed preferences [69].

Motion controls allow users to interact with virtual environments using their natural body movements. In combination with HMDs, motion control allows for designing game mechanics that rely more on such natural movements, spatial awareness, and binocular vision e.g. puzzles requiring viewing and manipulating objects from multiple angles [87]. Of the HMD-VR health games reviewed, nine relied on the headset alone. An equal number used skeletal tracking technology to capture movements of the player’s body and limbs to control the game. For physical exercise, five games used stationary cycles. A few games used hand motion controllers or optical hand tracking while two others involved robots for upper limb rehabilitation. These types of motion controls allow for more intuitive interactions with virtual game environments, compared to traditional gamepads or mouse and keyboard combinations [88]. However, of the games we reviewed, game design focused less on unique mechanics afforded by HMD-VR and more on its presence and immersion advantage. However, there remains a tension between designing interactions that are intuitive (e.g. using hand movements to manipulate levers on a machine) and simple and reliable (e.g. performing complex actions by pressing a controller button) [89]. Moreover, players’ familiarity with traditional game controls may impact their expectations and perceptions of the game [90]. Ultimately, these diverse features allow for VR applications that target a variety of health contexts. Moreover, they enable therapeutic methods that would otherwise require substantial physical resources (e.g. varied, dynamic, and highly controlled training scenarios) [91] or would not be possible (e.g. virtually exaggerated or reduced body movement) [92].

Cybersickness remains a persistent challenge in HMD-VR design considerations [14]. Indeed, individuals with a history of motion sickness or cybersickness were necessarily excluded in some studies reviewed [57, 74, 76]. Moreover, the consideration for cybersickness limits game design from certain ways of implementing game mechanics, especially with respect to moving through the virtual world [93]. Porcino and colleagues have proposed some design guidelines on how to reduce cybersickness, including minimizing field of view changes not in the player’s control and reducing the acceleration of movement [94]. In our review, Barathi and colleagues designed their cycling game to keep the player’s view fixed forward to minimize cybersickness. Participants who played *Lumapath* said static and clear geometric shapes with high contrast helped them feel grounded, however the HMD’s weight became uncomfortable. Yet, videogame players have also demonstrated a willingness to tolerate such usability disadvantages in exchange for a more immersive experience [89]. However, it remains unclear whether such attitudes persist in populations less familiar with videogames, or in clinical populations, where it may be exacerbated by other issues that make them more susceptible.

### Game design in HMD-VR health games

HMD-VR health games presented game design with a range of detail (Table 2); 8 of 29 games lacked sufficient detail to summarize in terms of mechanics, dynamics, and aesthetics. Meaningful play, which has been previously discussed in games for health [31, 32], was not mentioned at all, while only a handful of studies framed game design in terms of designing gameplay or mechanics [48, 51, 54, 55, 57, 60, 63]. In many studies, especially those where multiple small-scale games were involved, game design was typically described as a brief scenario in terms of game controls, thematic setting, primary activity, and game outcome, which was usually a score. For example, the lower limb rehabilitation game BeTheBall for post-stroke patients was described as a scenario where players “perform at a faster path motivated by his own high-score presented in the form of a ghost [by] maximizing accuracy and velocity” and included “different types of races and environments”. Such descriptions provide a rudimentary overview of the mechanics and aesthetics of the game, i.e. the rules of the game and how the designers want the player to feel while playing it. However, greater detail in the reporting of game design is required to understand how the game dynamics support moment-to-moment engagement of the player with the game. Being able to discern the actual strategies and approaches to game playing that are designed for players to enact will help us to better understand how particular game design choices work to support behavior change. In studies that did provide greater detail, few explicitly addressed game design on a theoretical level [48, 60]. Notably, one game was designed in consideration of the MDA framework [57].

As the majority of HMD-VR health games targeted physical exercise and motor rehabilitation, game design trends tended to follow the mechanics and aesthetics typical of exergames [95]. A common approach to designing the rules and structure of the VR game involved directly recreating the therapeutic task and applying points and scoring mechanics to the performance of this task. For example, cycling games for physical exercise typically tied to the power generated or distance travelled by the player to their score as they navigate obstacles [49, 55]. Indeed, points, scoring, or prize mechanics were specified in 17 games, with many emphasizing these mechanics as key sources of motivation. Rewards-based mechanics generally give rise to game dynamics involving time pressure and striving for the highest score, with an overall game aesthetic of improvement and accumulation by overcoming challenge. For example, both Cycling Obstacle Course [55] and Rift-a-bike [49] attribute player motivation to such reward mechanics. Some games recreate scenarios involving activities of daily living with controlled parameters, allowing for graded difficulty [53, 61]. Overall, these approaches to supporting motivation lean more towards “gamification” [96, 97] of therapeutic tasks, whereby game mechanics are implemented to serve primarily as feedback and reward, similar to that provided by a coach or therapist, and less to build interesting game dynamics and aesthetics. They have the advantage of being familiar, providing direct feedback for the task, and being clear in therapeutic relevance. However, as a games that rely heavily on extrinsic rewards (points and badges), they may be limited in sustainable engagement without attention given to crafting intrinsically rewarding game experiences [98]. Barathi and colleagues [57] discussed intrinsic motivation in their exercise game, though it was still in context of scoring and overcoming challenge. In games by Ijaz and colleagues, self-determination theory was cited as underpinning motivation for playing games [63]. However, they did not discuss how it was incorporated into game design.

Only three games involved narrative as a means of engaging end-users. For example, the upper-limb stroke rehabilitation game, “A Priest in the Air”, sets the player traveling through different countries in an air balloon to reach a king, using arm movements to guide the balloon into point-scoring objects [60]. Other games simply involved aesthetics in terms of thematic setting and goals [62, 70, 76]. One game used pedaling mechanics to navigate a city and capture unique creatures [62] while another used the mechanics of capturing a flying butterfly to elicit head movements for neck rehabilitation [66]. However, these games still relied primarily on “do more to score more” dynamics.

Conversely, a few game designs involved mechanics and dynamics that focused on aesthetics of sense-pleasure, discovery, and narrative. Gobron and colleagues used the mechanics of a robotic-pedal interface as piloting controls for a spacecraft [50]; in this way the mechanics directly supported embodiment of the game aesthetic. In Mobius Floe, high levels of interactivity and multisensory elements aimed to immerse the end-user in a wintry environment. Hostile objects and combat mechanics served metaphorical roles to involve the end-user in a symbolic narrative of overcoming pain [48]. In a user study of “LumaPath”, a movement-based exploratory game set in a fantastical world for individuals with arthritis, end-users emphasized that exploration was what kept them engaged as well as comfortable with learning the game [51]. The immersive advantages of HMD-VR directly support the design goal of creating these types of aesthetic experiences that focus on being present in a particular environment. In comparison, aesthetics focused on the achievement of high scores are less reliant on HMD-VR to be successful, as the experience of getting points and a high score can be equally effective when presented on a 2-dimensional display. As such, exergames focused on point-scoring may benefit from game design that employs mechanics and dynamics that better leverages the advantages of HMD-VR. The cycling game by Barathi and colleagues follows this route by creating dynamic moods of calm and urgency as the player fulfills the role of a bicycle delivery person weaving in and out of traffic [57]. At a smaller scale, multisensory design elements such as visual effects and audio cues were often pointed to specifically for providing feedback and lending sense-pleasure aesthetics: for example, particles emitting from a butterfly [66] or sounds accompanying reward collection and other events [60, 70].

Overall, the HMD-VR game design discourse for health application echoes that of game design related to non-immersive VR health applications, where the focus remains on using numerical feedback and reward systems to drive motivation and engagement [80]. For example, Burke and colleagues link meaningful play in games for stroke rehabilitation to performance feedback of the therapeutic task in the form of scoring or progress bars. Indeed, the majority of HMD-VR health games reviewed highlighted scoring points as a core mechanic.

Within the HMD-VR health games literature, there lacks a more thorough discussion of designing the game as a compelling game in-of-itself. From the games industry, one common way of discussing a game is in terms of its core loop: the primary repetitive series of actions that a player takes while interacting with the game [99]. This is the “heart” of the game and is made up of the most essential mechanics and dynamics of a game. For example, the core loop of soccer involves a cycle of positioning, dribbling, and shooting. Having a core loop that is in-of-itself compelling for players to engage with, is key to making games effective at supporting motivation and engagement with therapeutic tasks. It is what underpins the behavior change aspect of the intervention. Without creating a good core loop, one is left with an interactive virtual environment, but a potentially boring game.

Beyond a well-designed core loop, the duration of the whole health game experience should also be explicitly addressed, whether as a course of treatment spanning several sessions or something lasting years. Commercially, some games are designed to be played through once or twice, e.g. puzzle or story-driven games, while most games are designed to be played repeatedly without limit. Given the latter is an implicit objective of most health games as behavior change tools, replayability is a crucial consideration. While the concept of replayability has been touched on [32], it has not been featured as a fundamental design goal for successful health games. Nevertheless, the HMD-VR health games reviewed have used mechanics such as varied challenges, goals, and environments to support replayability [57, 74]. Cycling Obstacle Course [55] and A Priest in the Air [60] also involve decision-making through branching paths that can also support replayability. Other approaches in games industry include varied player abilities, player roles, or mechanics using randomization [100]. Oppositional games such as chess or soccer succeed in replayability by having game with large possibility spaces where players must choose amongst many approaches to playing the game and respond to their opponents’ choices. Ultimately, replayability relies on game dynamics that remain interesting for players to engage with over repeated play sessions. Some academic literature has aimed to provide guidance on designing for replayability [101].

Renowned game designer Sid Meier once defined games as a series of interesting decisions [102]. Certainly, the success of a game’s core loop or its replayability may be judged by how well it continuously provides players with such “interesting decisions.” Future development of HMD-VR health games may benefit from greater attention to these design considerations. Finally, a more detailed account of game design in future reporting may facilitate more holistic evaluation and adoption of these games in clinical practice.

### Evaluation and adoption of HMD-VR health games

Of the studies reviewed, 24 involved evaluation of HMD-VR health games while five studies were purely descriptive of the technology (Table 3). Moreover, six studies captured qualitative user feedback using open-ended questionnaire items or semi-structured interviews. For quantitative measures, therapeutic outcomes such as energy output for exergames or visual analog scale for pain reduction were evaluated for 17 games. While these outcomes primarily serve to measure clinical status and therapeutic efficacy, they also underpin how a given therapeutic approach is translated into the game format. In terms of game design, therapeutic outcomes are often incorporated into game mechanics such as scoring or adaptive game difficulty. For example, exercise games by Barathi and colleagues as well as Shaw and colleagues reported power output of and calories burned by the player [55, 57]. The benefit in this case is that players receive feedback that is obviously relevant to their progress in addressing their health goals. However, this approach should be used with caution, as therapeutic progress is sometimes discouragingly slow [103]. Moreover, the incorporation of therapeutic outcomes into game design should be consistent with and support game dynamics and meaningful play.

While therapeutic outcomes remain the principle indicator of a health technology’s overall usefulness, adoption by users is also crucial in determining the long-term viability of a technology [104]. Adoption captures the users’ inclination towards accepting and using an HMD-VR health game. Taking into account constructs contributing to adoption by users during the design and development process can support maximizing the adoption potential of health games. For example, qualitative feedback from older adults who played *Lumapath* highlighted a need for more guidance through the game experience to aid usability [51]. Indeed, while some of the reviewed studies relied on researchers and clinicians to provide guidance, others included instructions, videos, and in-game tutorials or practice scenes to support usability [48, 59, 60, 63, 71, 74].

Quantitatively, constructs of technology adoption were evaluated in 16 studies primarily using ad-hoc questionnaires. These included constructs such as satisfaction, motivation or willingness to use, or ease of use. Validated measures such as the System Usability Questionnaire [105] appeared in three studies and the Intrinsic Motivation Inventory [106] was used for only one game. Similarly, 4 studies measured cybersickness either with ad-hoc Likert items or using the simulator sickness questionnaire [107]. While ad-hoc questionnaires can be tailored for the unique design goals of a HMD-VR health game, using validated measures can aid in making comparisons between games. Moreover, technology adoption theory appears largely absent from the literature reviewed. For example, the Unified Theory of Acceptance and Use of Technology (UTAUT) is a popular model for comprehensively describing the antecedents of intention-to-use technology generally [108, 109]. Specifically, Huygelier and colleagues used the UTAUT to explore acceptance of HMD-VR by older adults [110]. Designing HMD-VR health games with consideration for adoption and usability theory may also serve to ensure any weaknesses of these games are attributable to their game design as opposed to limitations in usability.

Ultimately, the purpose of leveraging a game format is to support sustainable engagement with a therapeutic approach. As such, successful game design inherently supports adoption and serves as a means of behavior change for therapeutic adherence. In this respect, HMD-VR health games have been evaluated by how engaging an experience they are. For 12 games, gameplay experience was evaluated using ad-hoc Likert scale questionnaires, with occasional usage of more formal questionnaires such as the Game Experience Questionnaire [111] or Igroup Presence Questionnaire [112]. Overall, evaluation of gameplay experiences focused on constructs such as fun or enjoyment, immersion and presence, and game difficulty. Together, enjoyment and presence speak to how effectively the game involves the player in the aesthetic experience. Perceptions of game difficulty can indicate how well game dynamics engage or challenge players without frustrating them. However, measures of fun and enjoyment lack specificity in the multiple ways a game may succeed as an aesthetic experience. For example, participants who played LumaPath highlighted how exploration was what they liked most and aspects of the game that limited exploration detracted from their experience [51]. This is quite different from how a high intensity cycling exergame achieves an enjoyable aesthetic experience through challenge and extrinsic rewards [57]. Moreover, evaluation of gameplay experiences have typically involved impressions during or shortly after first encountering the game and with limited time to master game mechanics and dynamics. Currently, the field exists in a state of perpetual novelty. As the field of HMD-VR games for health continues to grow, understanding the qualities that distinguish more successful game design in the health context will require longer-term and aesthetic-oriented evaluations of gameplay experience.

### Stakeholder engagement

In order to improve the adoption potential of health technologies for patients, there have been increasing calls to incorporate stakeholder engagement throughout the technology development process, with particular emphasis on involving end-users [39, 43, 113]. For HMD-VR health games, key stakeholders not only include end-users, but also informal caregivers and clinicians. Stakeholder engagement in technology development encompasses a range of activities from basic user testing and feedback to more participatory or co-design oriented approaches [42].

Of the 29 HMD-VR health games reviewed, 4 indicated collaboration with clinicians for designing the game [60, 61, 65, 74]. However, details of the collaboration were not typically discussed in-depth. As indicated by usability and gameplay experience outcomes, the majority of articles involved various forms of user testing, with some providing opportunity for end-users to give feedback through interview responses. These approaches generally align with user-centered design, which emphasizes iterative prototyping and testing in order to ensure products are usable and useful for end-users [114]. However, user-centered design (UCD) was not explicitly indicated as an approach in all but one of the reviewed articles [74]. In non-HMD-VR health games, UCD has been a common development approach. For example, Brox and colleagues proposed a UCD protocol specific to designing exergames for older adults. In a review of health games (including non-HMD-VR) for anxiety and depression, UCD informed the design in 7 out of 20 games [115]. Beyond UCD, which has traditionally positioned end-users as purely informants, design in research settings have seen a shift towards more collaborative and participatory approaches such as co-design [116, 117].

Engaging end-users through participatory approaches, including the field of co-design, may be particularly beneficial in the context of HMD-VR, as this context relies on more dynamic human–computer interactions with practical issues that can make implementation challenging. More participatory approaches aim to empower stakeholders as equitable partners in designing technology and are characterized by mutual commitment, learning, and alignment with stakeholder values [118]. Participatory projects may use methods such as recurrent workshops and focus groups or involve stakeholders as full team members to meaningfully incorporate stakeholder perspectives in design decisions [116]. For example, future development of games such as *Lumapath* may involve older adults throughout the design process to help address topics such as guidance, input simplicity, or further capitalizing on exploration as the main motivating factor, rather than identifying such considerations in later testing. As such, participatory approaches may support a more democratic method of identifying and valuing person-centered considerations regarding usability, playability, and therapeutic value early and throughout the design process of HMD-VR health games.

Of the studies reviewed, only one described engagement with end-users in the design process beyond a purely informant capacity. Eisapour and colleagues followed a participatory design approach involving both clinician users and end-users with mild cognitive impairment [74]. Their approach used focus groups and user tests to collaboratively decide on thematic setting, therapeutic exercises to include, and implementation of interactions. This process aided the authors in prototyping an exergame that aligned with end-users’ preferences and addressed hidden problems early on. This example is consistent with other participatory studies in similar health technology applications. In non-gaming HMD-VR health applications, participatory methods have been used to prototype exposure therapy scenarios [119] and stress reduction treatment for teens [120]. Similarly, Webster and colleagues developed a hand rehabilitation game with people with multiple sclerosis [121]. Ultimately, participatory approaches may facilitate better game design at all levels, in fine-tuning mechanics, designing satisfying dynamics, and identifying aesthetics that resonate most strongly with a population.

### Limitations

The search strategy included articles explicitly using HMD and VR as specific terms. As such, we may not have captured articles using only the general term “virtual reality.” However, we deemed this strategy to have much greater specificity and identifies the most relevant articles with substantive discussion of HMD-VR.

While a narrative approach is appropriate for the range of game design descriptions present in the articles reviewed, this approach is more interpretive than systematic or scoping reviews. For example, game design summaries required more interpretation where more design details were missing. As such, the findings of this review should be received accordingly. Nevertheless, the narrative approach allows for greater flexibility in situating the particular characteristics of HMD-VR health games within the nuanced context of health technology and game design.

## Conclusions

HMD-VR health games is a relatively new and growing field of tools for engaging clients in highly immersive games designed to address health contexts ranging from rehabilitation exercise to pain management to sensory disorders. Our review provides a holistic overview of the prevailing trends in designing HMD-VR health games, including health context, game design, technology implementation and adoption, and user engagement in the development process. Overall, HMD-VR health games typically involve obstacle-based challenges, points, and extrinsic reward systems to engage clients in interventions primarily focusing on health contexts related to physical functioning and pain. The technology used to implement these games reflects this trend, with many using skeletal tracking and stationary cycles. Less common were games with emphasis on narrative experiences and use of hand motion controllers, which may better align with non-physical exercise interventions. However, the extent to which game-based formats (versus non-game formats) or HMD-VR technology is most appropriate for less represented health contexts such as mental health requires more research. Nevertheless, we anticipate further growth in diverse and complementary HMD-VR health games.

As HMD-VR technology continues to rapidly evolve, the opportunities and ways in which such games can address health contexts will continue to grow. However, these applications will continue to rely on fundamental principles of game design. Indeed, there is a reciprocal relationship between HMD-VR technology creating new design spaces for games and game design needs advancing HMD-VR technology. Given the complex intersection of this relationship with health needs, the associated game design discourse is often lacking. More in-depth and structured attention to how HMD-VR health games are designed as game experiences is needed. This will support greater understanding of what design strategies are most effective for achieving therapeutic goals, including adherence. Finally, future development HMD-VR health games may benefit from more application of participatory approaches such as co-design, that involve end-users throughout the development process. Successful alignment of these games with end-users’ needs and values ultimately maximizes the impact of health games by facilitating their adoption.

## Supplementary Information

**Additional file 1: S1**. Datasheet.

## Data Availability

Not applicable.

## Abbreviations

HMD: Head-mounted display
VR: Virtual reality
UCD: User centred design
UTAUT: Unified theory of acceptance and use of technology
MDA: Mechanics-dynamics-aesthetics framework
